# Wnt-5A/B Signaling in Hematopoiesis throughout Life

**DOI:** 10.3390/cells9081801

**Published:** 2020-07-29

**Authors:** Marina Mastelaro de Rezende, Giselle Zenker Justo, Edgar Julian Paredes-Gamero, Reinoud Gosens

**Affiliations:** 1 Departamento de Bioquímica, Universidade Federal de São Paulo (UNIFESP), São Paulo 04044-020, Brazil; marina.mrez@gmail.com (M.M.d.R.); giselle.zenker@gmail.com (G.Z.J.); paredes.gamero@gmail.com (E.J.P.-G.); 2 Department of Molecular Pharmacology, University of Groningen, Groningen 9713 AV, The Netherlands; 3 Departamento de Ciências Farmacêuticas, Universidade Federal de São Paulo (UNIFESP), Diadema 09913-030, Brazil; 4 Faculdade de Ciências Farmacêuticas, Universidade Federal de Mato Grosso do Sul, Campo Grande 79070-900, Brazil

**Keywords:** hematopoiesis, noncanonical Wnt, Wnt5a, Wnt5b

## Abstract

Wnt signaling is well-known to play major roles in the hematopoietic system, from embryogenesis to aging and disease. In addition to the main β-catenin-dependent pathway, it is now clear that Wnt5a and the structurally related Wnt5b are essential for hematopoiesis, bone marrow colonization and the final steps of hematopoietic stem cell (HSC) maturation via β-catenin-independent signaling. Wnt5a and Wnt5b ligands prevent hematopoietic exhaustion (by maintaining quiescent, long-term HSCs), induce the proliferation of progenitors, and guide myeloid development, in addition to being involved in the development of aging-related alterations. The aim of this review is to summarize the current knowledge on these roles of Wnt5a and Wn5b signaling in the hematopoietic field.

## 1. Introduction

**Figure 1:**
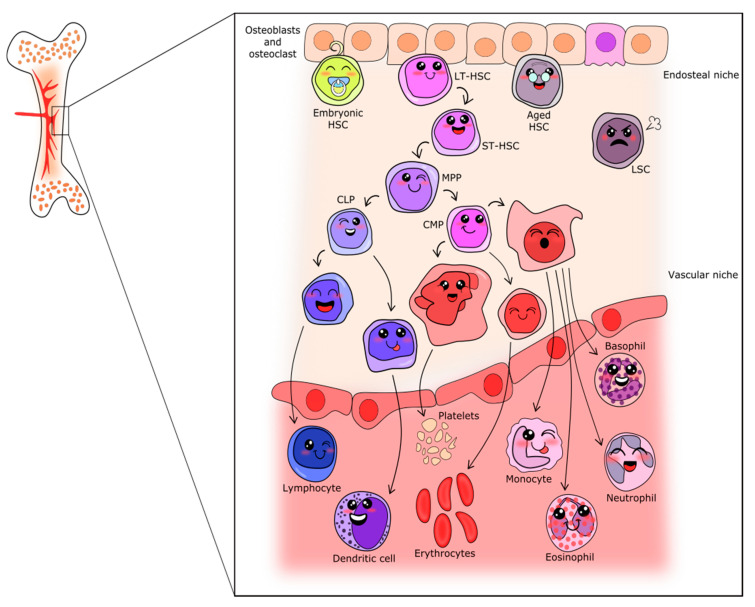
Schematic representation of the classical hierarchic model of hematopoiesis. From the endosteal niche (upper part of figure) to the blood vessel, passing through the vascular niche, the following distinct populations are depicted: LT-HSC—Long-term hematopoietic stem cells; ST-HSC—Short-term hematopoietic stem cells; LSC—leukemic stem cells; MPP—Multipotent progenitor; CLP—Common Lymphoid progenitor; CMP—Common Myeloid progenitor; and the cells between the committed progenitors and mature cells.

Hematopoiesis is the process in which all blood cells are produced, starting from a unique and scarce population of hematopoietic stem cells (HSCs). This population is characterized by multipotency (ability to form mature cells of various lineages of same tissue), capacity for self-renewal, and long-term hematopoietic maintenance after transplantation [1,2]. As represented in Figure 1, it is well accepted that HSC stands in the upper position of a hierarchic system that relies on proliferation and differentiation [1,3,4]. In fact, the clonal potential of HSC was the key point for stem cell research initiation [4]. In adults, HSCs rarely enter the cell cycle [5], however, when they do, their fate may change, being followed by gradual potential loss [2]. In this context of HSC cycling, three possible pathways are possible: (1) Symmetric division without differentiation or self-renewal, characterized by cell division without transcriptional alterations that would lead to lineage commitment. This represents an important process to increase or maintain the undifferentiated pool of HSCs and avoid HSC exhaustion [3,6,7,8]. (2) Symmetric division with differentiation, characterized by the production of two daughter cells, each harboring slightly less multilineage potency but exhibiting an increased proliferative index. These are the so-called multipotent progenitors. This process is essential in case of stress and when in acute need of mature cells [3]. (3) Asymmetric division, characterized by the uneven production of daughter cells, one similar to the mother (stem) cell and one multipotent progenitor [3,8].

According to the classical model of hematopoietic differentiation, the multipotent progenitors separate into two branches from which myeloid or lymphoid lineages will arise [9,10]. These cells will enter the cell cycle and differentiate as needed to achieve mature blood cell production [11]. Differentiation is accomplished by the accumulation of transcriptional changes, resulting in properties and functions gains, as well as changes in immunophenotyping profile, which is used as a hallmark for each differentiation step. Cell surface markers associated with each illustrated cell are shown in Supplementary Table S1. The myeloid branch drives the formation of platelets, erythrocytes, monocytes, and granulocytes (neutrophils, eosinophils and basophils), whereas the lymphoid branch drives the formation of lymphocytes and natural-killer cells. Figure 1 summarizes the events classically involved in hematopoiesis.

Although the classical model is well accepted for adults and illustrates the steps involved in hematopoiesis, it is noteworthy that numerous other models were proposed and are often being revised to include new findings, such as the uneven time point in progenitors branching from HSC [2,12,13,14,15,16,17] and the view in which differentiation is a continuum of transcriptional changes [18,19], with progenitors having heterogeneous potential [1,12,18], as well as some mature cells having multiple progenitors [20]. Furthermore, the classical view does not include alternative differentiation pathways for HSC [1,16].

It is known that cytokines and growth factors are key players in all steps of hematopoietic regulation and development, but new factors in this context are not commonly described. Recent findings point to an important role for Wnt signaling in HSC maintenance and differentiation, showing that the Wnt pathway is crucial to subsequent events in myeloid and lymphoid differentiation. Whereas, originally, Wnt/β-catenin-dependent signaling was a main area of focus, it is now clear that signaling through β-catenin independent signaling with the involvement of Wnt5a and Wnt5b plays major roles in hematopoietic development, differentiation and ageing. The aim of this review is to summarize the current knowledge on the role of Wnt5a and -b signaling in the hematopoietic field. Here, when HSC are addressed, this refers to ST-HSC, except when specified to besomething else.

## 2. Wnt Signaling

Wnt ligands are secreted lipid-modified glycoproteins, which rely on their post-translational modifications for the secretion and activation of their receptors (glycosylation and palmitoylation, respectively) [21,22]. The hydrophobic portion of the Wnt ligand binds to the cysteine-rich domain of the N-terminus of a group of receptors, referred to as frizzleds (Fzds), which are localized in the plasma membrane [23,24]. The binding of a Wnt ligand to the Fzd receptor at the cysteine-rich domain activates the receptor. There is little variation in the cysteine-rich extracellular portions of Fzd receptors. In addition, the post-translational modifications present in Wnt ligands are also similar, which explains the high degree of promiscuity in the binding of Wnt ligands to Fzd receptors [24,25]. 

**Figure 2:**
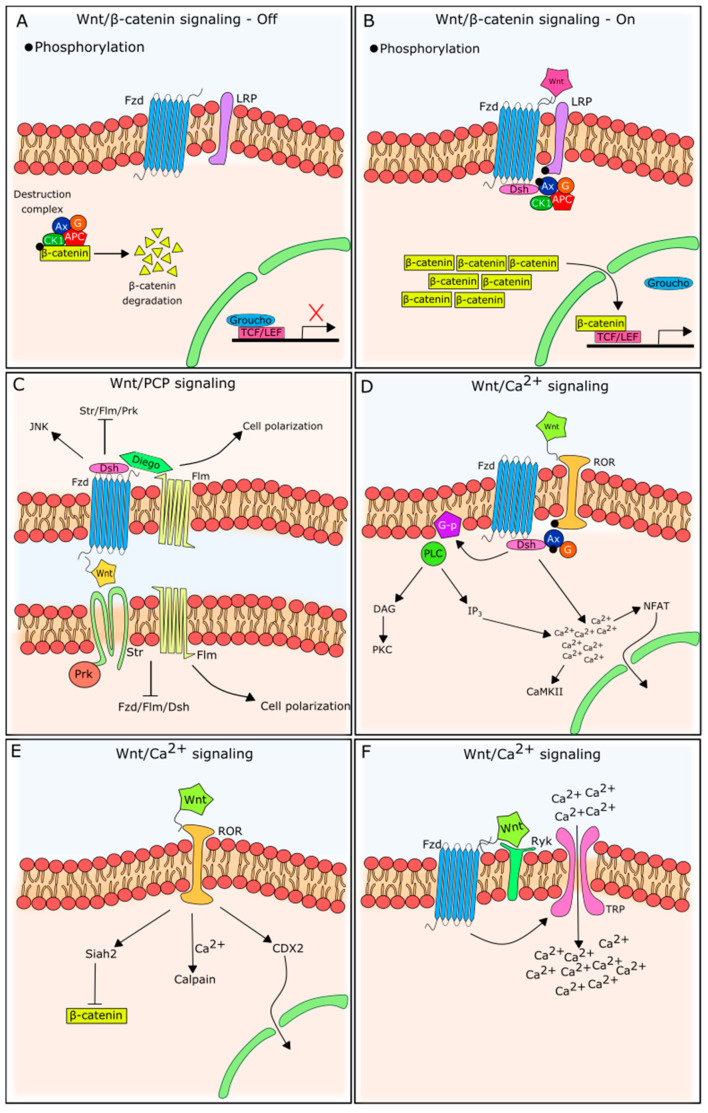
Wnt signaling scheme. (**A**) Wnt/β-catenin dependent signaling in its inactive status. There is no Wnt ligand and receptor binding, so the destruction complex targets β-catenin, routing it for degradation. Expression of TCF/LEF genes is inhibited by Groucho. Fzd—Frizzled receptor; Ax—Axin; G—GSK-3β; CK1—casein kinase 1; APC—adenomatous polyposis coli. (**B**) Wnt/β-catenin dependent signaling in its active status. After Wnt ligand and Fzd receptor binding, in the presence of LRP, the destruction complex is attracted to the plasma membrane region by Dsh action. β-catenin is not degraded and accumulates in the cytoplasm and nucleus, activating transcription of TCF/LEF genes. Dsh—Dishevelled. (**C**) Wnt/PCP signaling is independent of β-catenin and relies on asymmetric distribution of membrane proteins. At one cellular side, there is Dsh recruitment as well, however other proteins are targeted, such as Diego and Flm (Flamingo). At the other cellular side (and in the neighboring cell), Prickle (Prk) and Strabismus (Str) will be present, in addition to Flm. Although Flm and Str are well accepted nomenclatures in *Drosophila*, in mammals, they are known as Celsr and Vangl, respectively. These proteins interact internally and with neighboring cells, regulating cell polarity. (**D**–**F**) Wnt/Ca^2+^ signaling variations. (**D**) Wnt/Ca^2+^ signaling by ROR action as a Fzd co-receptor, and G-protein and PLCβ activation, which leads to Ca^2+^ release from intracellular stores. PLC—Phospholipase C; G-p—G-protein; DAG—diacylglycerol; PKC—protein kinase C; IP3—inositol trisphosphate; CaMKII—calmodulin kinase II; NFAT—Nuclear factor of activated T-cells. (**E**) Wnt/Ca^2+^ signaling in the absence of Fzd and in the presence of ROR. (**F**) Wnt/Ca^2+^ signaling in the presence of Ryk co-receptor and an increase in intracellular Ca^2+^, induced by TRP activation. TRP—transient receptor potential.

The β-catenin pathway has been the most described and studied, being triggered when a Wnt ligand binds to a Fzd receptor in the presence of low-density, lipoprotein-receptor-related protein (LRP)5/6 co-stimulatory receptors [26,27]. β-catenin-independent signaling pathways, on the other hand, are triggered by Wnt ligand–Fzd receptor couples, but in the presence of different co-receptors, such as receptor Tyrosine Kinase Like Orphan Receptor 2 (ROR2) and receptor-like tyrosine kinase (RYK), in an LRP5/6-independent manner [28,29]. Both are represented in Figure 2.

### 2.1. β-Catenin-Dependent Wnt Signaling

In the absence of an extracellular Wnt ligand, the cytoplasmic abundance of β-catenin is kept low due to phosphorylation, ubiquitination, and proteasome degradation [30]. This is the result of a destruction complex in the cytoplasm, in which Axin clusters with APC, CK1, and GSK-3β to target β-catenin, marking it for destruction.

When an extracellular Wnt ligand couples to its Fzd receptor, it triggers intracellular alterations that induce the stabilization of β-catenin. For Wnt/β-catenin signaling, the Fzd receptor has to be juxtaposed with an LRP family molecule. LRP is recruited in the presence of Wnt, forming a heterotrimeric complex responsible for intracellular signaling [26,31]. LRP phosphorylation recruits and binds to Axin in the cytoplasmic tail [32], together with Dishevelled (Dsh). The migration of Axin from the cytoplasm to the plasma membrane inactivates the destruction complex. Accordingly, cytoplasmic β-catenin is no longer routed to the degradation pathway. The cytoplasmic accumulation of β-catenin induces its molecular migration to the nucleus [31]. 

β-catenin activity in the nucleus is normally inhibited by gene repressors (the most well-known is Groucho), but its accumulation competitively drives these repressors away from transcriptional target sites, allowing TCF and LEF-1 DNA-binding and gene transcription, stimulated by β-catenin [31]. It is important to note that the transcriptional outcome of Wnt/β-catenin pathway activation varies with cell type [33]. Although Wnt3a is the prototypical ligand that unleashed β-catenin-dependent Wnt signaling activation [34,35], it is referred to as Wnt3 or Wnt3a, depending on the nomenclature used by the referred article.

### 2.2. β-Catenin-Independent Wnt Signaling

In addition to Wnt/β-catenin signaling, Wnt ligands can trigger several β-catenin-independent pathways (often referred to as noncanonical signaling). As observed for Wnt/β-catenin signaling, these pathways are activated by extracellular Wnt ligand binding to a plasma membrane receptor, triggering intracellular changes. In vertebrates, β-catenin-independent Wnt signaling is primarily categorized as two main pathways: PCP and Wnt/Ca^2+^ [36], although further subdivisions have been described [37].

#### 2.2.1. Wnt/PCP Signaling

The planar cell polarity (PCP) pathway, first identified in Drosophila, is the best characterized β-catenin-independent pathway, and is essential in cellular polarity. As for the Wnt/β-catenin pathway, the PCP pathway is widely conserved evolutionarily and uses Fzd receptors, but without the need for LRP molecules [38].

The PCP signaling pathway is involved in the directional instruction of cells in a tissue to support its functional properties. In the first studies on Wnt/PCP signaling [39,40], Fzd receptors were promptly identified as players in the epithelial orientation. The main tissues (in Drosophila) in which PCP signaling was first discovered were the epidermis and the wing, both covered by hair that needs to be oriented in the right direction in order to work properly [38].

Although the Wnt/PCP pathway shares the use of the Fzd receptor with β-catenin-dependent signaling, the organization of these molecules in the plasma membrane differs markedly, as can be seen in Figure 2. First of all, its asymmetrical distribution in the plasma membrane and lack of involvement of LRP molecules are unique to this pathway [41]. Moreover, Fzd receptors join a membrane complex—the PCP/core complex—with five other proteins (Flamingo, Strabismus, Dishevelled, Diego and Prickle) [38]. Ryk, acting as an Fzd co-receptor, may also be present and participate in complex stabilization and JNK activation [42,43,44]. Notably, although Flamingo and Strabismus proteins are well accepted nomenclatures in *Drosophila*, in vertebrates, they are known as Celsr and Vangl, respectively (as reviewed by Goodrich and Strutt [45]).

Interestingly, the asymmetrical distribution of these complexes forms an axis that orientates cellular function. The Wnt/PCP pathway is also known as the Wnt/JNK pathway [46], because of the downstream activation of JNK after Wnt5a binding to Fzd2.

#### 2.2.2. Wnt/Ca^2+^ Signaling

Unlike the PCP pathway, the Wnt/Ca^2+^ pathway is widely active in the hematopoietic system and has been a topic of increasing interest in recent years. The proposed pathway involves the activation of a G-protein after Wnt/Fzd binding [47] and results in PLCβ (phospholipase C beta) activation, Ca^2+^-release, CaMKII (calcium-calmodulin kinase II) and PKC (protein kinase C) phosphorylation [48,49]. The activation of NFAT transcription factors is one of the Wnt/Ca^2+^ signaling events driving subsequent gene transcription [50].

For this pathway, the above-mentioned co-receptor ROR2 plays a key role. ROR2 functions as a co-receptor for Fzd, and induces membrane PLCβ activation and second messenger production, such as inositol 1,4,5-trisphosphate (IP3) and 1,2 diacylglycerol (DAG), which results in Ca^2+^ release from the endoplasmic reticulum, leading to increased cytoplasmic Ca^2+^ concentrations [51]. When Wnt5a interacts with ROR directly, Siah2, calpain or CDX2 can be activated [51], which may inhibit β-catenin signaling, providing a means for the Wnt/β-catenin-independent pathway to counteract Wnt/β-catenin-dependent signaling.

Ryk has also been associated with Wnt/Ca^2+^ signaling, involving two possible mechanisms: (1) activation of G-proteins (similar to ROR2), and (2) activation of transient receptor potential (TRP) receptors, causing extracellular Ca^2+^ influx [52].

## 3. Wnt Signaling in Hematopoietic Ontogenesis

Studies on Wnt signaling in the hematopoietic field started a few years after the initial discovery of Wnt and were mainly focused on the β-catenin-dependent branch. Wnt signaling appears to play key roles in the ontogenesis of the hematopoietic tissue, from the embryonic period to adult stages.

### 3.1. Hematopoietic Development

The primitive endoderm and primitive streak are particularly important in the ontogeny of the hematopoietic system, since they are precursors of the yolk sac, in which the first wave of hematopoietic production takes place (primitive hematopoiesis) [53]. However, the context for HSCs’ first appearance [54], the potential of these HSCs [55] and how they are related to adult HSCs is still controversial [56]. In this context, it is noteworthy that other cells than HSC also seem to have roles in hematopoietic cell production, as described for tissue macrophages [57,58].

Yolk sac and blood island formation is crucial in hematopoietic initiation and, at this point, Wnt5a gene expression peaks and its signaling appears to play an important role. Interestingly, this initial hematopoietic commitment can be accelerated by DMSO treatment and repressed by retinoic acid [59]. Accordingly, DMSO treatment stimulates Wnt5a expression, while retinoic acid decreases it [60]. Of note, retinoic acid increases the expression of Wnt/β-catenin-dependent Wnt3a. This supports the idea that Wnt5a is involved in hematopoietic initiation through β-catenin-independent signaling [59], although Wnt5a is not indispensable, as Wnt5a knockout mice develop a hematopoietic system [61]. Interestingly, Wnt5b shows a similar expression pattern to Wnt5a, peaking during the first steps and showing reduced levels when hematopoietic commitment and proliferation transpire [60].

Studies using embryonic stem cell lines describe stage-specific waves of Wnt (the in vivo relevance of this is not established). In these cells, right before the formation of embryonic bodies (a stage that resembles the embryonic three germ layers), there is a peak in Wnt5a expression that seems to be related to hematopoietic initiation, reflecting the observations in vivo. Embryonic bodies can differentiate to blast-like colonies, immunophenotypically expressing Sca-1 and c-Kit (CD117), recognized markers of primitive hematopoietic development [59]. At this point of differentiation, at which hematopoietic cells are ready to start primitive hematopoiesis, the expression of other Wnt ligands peaks (Wnt3, 4, 5a, 5b, 7a and 8a), which seems to be linked to hematopoietic development [59]. Myeloid and lymphoid lineage cells can be obtained from these blast-like colonies, since embryonic blast colonies are pluripotent [62]. The induction of differentiation and lineage specification can occur by stimulation with growth factors, following peaks in Wnt3, Wnt6 and Wnt16 expression, as described. Interestingly, these Wnt ligands have also been observed in vivo during hemangioblast formation [59]. Hemangioblasts and blast-like colony structures are supposed to be the precursors of the hematopoietic system and stem cells, as they have endothelial and hematopoietic roots [59]. Thus, these data imply specific roles for Wnt3 and Wnt5a signaling during early hematopoiesis. The specification and formation of early hematopoiesis from the undifferentiated mesoderm seems to be dependent on Wnt5a, whereas the expansion and commitment of hematopoietic-programmed cells might be Wnt3-dependent [60]. Interestingly, in fetal life, contrary to what is observed in adults, the HSC pool is described as promptly used to maintain the first production of immune and blood cells [63], which hampers the understanding of the roles of these cells in the adult HSC development.

As discussed previously, it may be too simplistic to extrapolate pathway activation only on the basis of ligand gene expression [59]. LRP5 was also investigated and its gene expression seems to have a stage-specific pattern. LRP5 gene expression peaks at early primitive hematopoietic development, at the same time as Wnt5a and Fzd4 gene expression [59], which might indicate a Wnt5a role in β-catenin-dependent pathway activation. High LRP5 gene expression is observed again in the last stages of hematopoietic development, when blast-like colonies (in vitro) or hemangioblasts (in vivo) form the primitive organs of blood production and the HSC pool proliferates [59]. Regardless of some peculiarities between in vitro and in vivo observations, it can be hypothesized that Wnt5a, through β-catenin-independent signaling, participates in mesoderm differentiation, leading to hematopoietic initiation.

### 3.2. Definitive Hematopoiesis

Definitive hematopoiesis originates in the fetal liver (although HSCs can be observed in the placenta, yolk sac, and the aorta-gonad-mesonephros region) with the colonization of progenitor cells, and subsequently, after maturation, migration to the spleen and bone marrow [64]. Bone marrow engraftment dictates the environment and relationships that will be maintained throughout its life and it is described as also affecting HSC potential and heterogeneity balance [55]. The most relevant changes in the hematopoietic cells in these last steps of development are related to their cell cycle status. In fetal liver, the cells are actively cycling and proliferating [65], which seems to be induced by Wnt3a and (probably) activation of the β-catenin pathway.

Studies in zebrafish showed that there is a peak in Wnt16 expression, a β-catenin-independent pathway activator, as soon as the cells arrive in the bone marrow [62,66]. It seems that this peak in pathway activation is linked to a final development in hematopoiesis and helps the cells to gain function and work as true stem cells—with low proliferation, self-renewing and multi-potent [62]. Wnt16 commonly exhibits similar effects to Wnt5b, so one would anticipate that Wnt5b might also be responsible for this last step of maturation. This hypothesis is reinforced by the fact that the loss of either ligand (Wnt16 or 5b) is associated with serious hematopoietic development complications [62], such as malformations and defects in HSC specification [62].

After bone marrow colonization, a flow of hematopoietic cell production is established, which is maintained for most of life, even though the fetal origin of adult HSCs is more accepted for lymphopoiesis than myelopoiesis [56]. Primitive cells are restricted to a specific locus in the bone marrow niche, most of them maintaining quiescence, with the progenitors forming all mature blood cells [12,63], as represented in Figure 1.

## 4. Adult Hematopoiesis

In addition to the HSCs and progenitors themselves, the microenvironment and extracellular molecules are important to hematopoiesis as well [64]. Ca^2+^ concentration is increased in peripheral bone areas and cells expressing membrane Ca^2+^ sensors are attracted to these areas, characterized by reduced irrigation and hypoxia [67,68]. In these endosteal niches, HSCs reside, most of them being quiescent and rarely entering the cell cycle [69].

The other bone regions, more central and irrigated, have a decreased concentration of extracellular Ca^2+^, but a higher presence of blood vessels and, as a consequence, are normoxic. The more active HSCs and multipotent progenitors reside in these vascular niches (Figure 1). Other niches (perivascular) were described as being close to blood vessels and also seem to maintain HSC [69].

This organization and pattern is maintained until death, changing only under pathologic conditions. The active HSC pool is responsible for the production of progenitors and mature cells, and as such serves as a pool for when these cells are needed. Meanwhile, the cells in the endosteal niche act as a reservoir of primitive cells and protect against hematopoietic exhaustion, entering the cell cycle only when necessary [5,70]. These populations have different engraftment potentials after transplant, being divided into short-term (ST-HSC) cells with reduced hematopoietic reconstitution potential, and long-term stem cells (LT-HSC), capable of broad lineage reconstitution for extended periods of time [71].

The differentiation and proliferation in the hematopoietic system follow a hierarchic model (as represented in Figure 1), in which LT-HSCs give rise to ST-HSCs. In turn, these cells generate multipotent progenitors, which start to gain characteristics and functions and commit to myeloid or lymphoid lineages. With progenitor commitment, successive steps of cell cycling and differentiation occur until maturation is complete, ultimately resulting in the formation of highly specialized blood cells.

Analysis of whole bone marrow revealed gene expression of Wnt2a, 2b, 3a, 5a and 10b [72,73,74,75,76,77], but this included cells forming the microenvironment, which might be responsible for the largest portion of the signal, as they are more prevalent—in fact, mesenchymal stem cells are described to be an important source of environmental Wnt5a [78] and a possible target for Fanconi Anemia treatment, exactly by its Wnt5a production [79]. Interestingly, Wnt5a is continuously expressed, even when profound environmental changes occur—such as in vitro cultivation—which is not observed for Wnt3a or 10b [74,75]. This may point to the hematopoietic origin of Wnt5a, although Wnt5a expression is also seen in niche-forming cells. In fact, in the hematopoietic system, Wnt5a seems to be the only Wnt gene broadly expressed in a variety of cells, including stem cells [72,73,80,81], progenitors [80,82], and B-cells [80]. All these data highlight a possible role for Wnt5a, challenging previous assumptions that this protein would have limited roles in adult hematopoiesis [83].

In addition to Wnt ligands, hematopoietic cells express a wide range of Fzd receptors; however, some seem population-specific, such as Fzd4. Indeed, expression of this receptor is distinct in primitive populations (higher in LT-HSCs in comparison with ST-HSCs and progenitors) [84], and is linked to the activation of Wnt5a signaling, suggesting a role for Wnt5a on LT-HSC. Additionally, other Wnt5a target receptors, such as Fzd8 [85], Ror2 [86], Ryk [80] and Flamingo [85], are also expressed on these cells, reinforcing the importance Wnt5a in this population.

Flamingo is commonly associated with the Wnt/PCP pathway, whereas Fzd4, Fzd8, Ror2 and Ryk seem more related to the Wnt/Ca^2+^ pathway. It can be hypothesized that Wnt/Ca^2+^ signaling would be important for the LT-HSC population, considering the increased levels of this ion in the endosteal niche, and the little available evidence for nuclear β-catenin accumulation in these cells.

The intracellular mechanisms involved in Wnt5a-driven quiescence maintenance are not completely elucidated, but Ryk participation and a consequent decrease in reactive oxygen species formation seem important [80], in addition to the modulation of Cdc42 activity [81]. The involvement of other intracellular pathways, such as PI3K/Akt [87], also indicate β-catenin pathway inhibition [75], and the inhibition of lipid raft clustering [87]. Interestingly, an increase in engraftment potential was observed after Wnt5a treatment [76,88]. An increase in homing ability was excluded as a possible cause [75], so it seems that the effects of Wnt5a are indeed related to maintenance of the cells in G0. It is possible that the results described by Florian and colleagues [81] were obtained on a slightly differentiated population, which presents reduced levels of Ryk, thus the effect of this receptor was diminished. Aside, the lack of stroma can influence stem cell responses, regardless of being a well-known driver of differentiation, as they themselves discussed.

For the ST-HSC, however, cell cycle activity as well as nuclear β-catenin accumulation is observed [81,89]. Furthermore, the membrane receptors and co-receptors display different patterns with differentiation. In this context, Wnt5a effects change drastically [88]. During differentiation, gradual changes are acquired and they lead to Wnt5a-driven nuclear β-catenin cytoplasmic accumulation as a consequence of membrane alterations—possibly by Fzd and LRP intermediation [86]. Alternatively, with Ryk reduction, reactive oxygen species are formed, which can also induce β-catenin signaling [80].

For this population, Wnt5a now acts as a growth factor and, instead of inhibiting, it promotes cell cycle progression [77]. Increasing responsiveness to Wnt3a has been described following differentiation, and we hypothesize that there is an overlap between Wnt3a and 5a responsiveness in targeting β-catenin-dependent signaling in ST-HSCs. This idea is in line with the previous literature in which Wnt3 is considered important for hematopoietic cell fate decision [60].

Additionally, Wnt5a also seems to have a role in reducing proliferation by inducing apoptosis in LT-HSCs [75], which may be lost downstream in the differentiation process, as progenitor cells present a high degree of proliferation. Indeed, increased colony formation of CD117 negative hematopoietic cells was observed after treatment with a Wnt3a-conditioned medium in colony formation assay [89], whereas Wnt5a treatment or Ryk inhibition had no effect on colony formation in this setting [80], in agreement with our hypothesis.

With hematopoietic branching in myeloid and lymphoid lineages, Wnt receptor and co-receptor expression patterns seem to change drastically and, as a consequence, responsiveness to one or several ligands may change as well. Knockout models for Wnt signaling were of great help to clarify the involvement of these pathways in hematopoiesis. For the lymphoid branch, Wnt5a knockout mice present decreased thymic cellularity and higher apoptosis of double positive thymocytes [71], coinciding with β-catenin nuclear accumulation, which suggest some role for Wnt5a in β-catenin pathway regulation, although long-term cellular responses to Wnt5a stimulation were absent [77].

Lymphoid Enhancer Binding Factor 1 (LEF-1), an important transcription factor for lymphopoiesis [90], is transiently expressed in specific stages of B lymphocyte differentiation [74], which may be linked to the activation of β-catenin-dependent signaling. Whether this involves Wnt5a is currently unclear.

For myelopoiesis, on the other hand, some roles for Wnt5a have been described [35], including its involvement in the specification of progenitors favoring the myeloid rather than the lymphoid branch [91,92]. Even for Wnt5b, there is evidence of significant roles in myeloid regulation, depending on the growth factors present [93].

Myelopoiesis is responsible for the formation of granulocytes, monocytes, platelets and erythrocytes, originating from a common myeloid progenitor, as schematized in Figure 1. In common myeloid progenitors, Wnt5a, as well as Wnt2b and 10b, are active, having similar roles and promoting proliferation [73].

Erythroid progenitors are highly responsive to Wnt5a, entering the cell cycle and proliferating (by PI3K activation) [94] or differentiating, giving rise to erythrocytes [95]. For thrombopoiesis, Wnt5b appears to have more effects than Wnt5a. In this case, G-protein signaling and its modulators seem to be involved, as its knockdown (of the G-protein signaling) caused thrombocytopenia in a zebrafish model [96]. The involvement of Wnt/Ca^2+^ signaling was proposed [96].

Little is known about Wnt5a (or b) in monocyte differentiation. Stimulation with Wnt5a had similar effects as Wnt3a treatment regarding IL-3 presence, causing an inhibition of monocytic differentiation [46]. Interestingly, there is evidence that environmental molecules may modulate Wnt5 effects, mainly Wnt5b, on monocyte differentiation [93].

Interestingly, granulopoiesis seems less affected in Wnt5a and -11 knockdown models [95]. It is dependent on LEF-1 expression, suggesting β-catenin-dependent pathway participation, as described for neutrophil differentiation [97]; little information on other types of granulocytes is available in this regard. For neutrophils in particular, Wnt5a is described to contribute to its migration and proinflammatory activity [98].

## 5. Hematopoiesis during Aging

There is increasing evidence that well-known hematopoietic alterations during aging (lymphoid to myeloid skewing, inflammation and decreased immunologic responses) are related to changes in Wnt signaling. The scenario for adult subjects seems to pass through gradual changes, in which the expression levels of environmental Wnt5a decrease, whereas the opposite takes place in HSCs, which acquire more Wnt5a with age [81]. These gradual changes were described already for middle-aged subjects, in which increased levels of Wnt5a, as well as Wnt4, were present in the primitive hematopoietic population [81], but without any clear functional outcomes. Interestingly, the described changes are restricted to the primitive pool and Wnt5a and 4, without significant changes in other populations or other Wnt ligands [81]. 

The increase in Wnt5a signaling activation in aged HSCs is proposed to inhibit HSC cell cycle entering and hamper hematopoietic differentiation, and it seems to affect both, LT and ST-HSC [81]. In addition, the balance between proliferation and apoptosis, described in ST-HSCs and due to the activation of β-catenin signaling, might be disrupted, which could account for the lower HSC replacement.

For the cells in the committed progenitor phase, this change in Wnt status may act to skew the lineage commitment in favor of myeloid cells, as lymphoid cells are more susceptible to Wnt5a mediated inhibition than myeloid cells. In fact, for the myeloid lineage, the increase in Wnt5a does not hinder differentiation; instead, it stimulates it, since granulocyte–monocyte progenitors as well as granulocytes are increased in aged subjects [91].

Interestingly, analyzing aged mice haploinsufficient for Wnt5a (Wnt5a^+/−^) provides more evidence that Wnt5a is indeed one of the main molecules related to aging-related myeloid skewing, since these animals do not present increased Wnt5a expression with aging and their blood cell counts are similar to young control animals [81].

Evidence suggests that Wnt5a triggers β-catenin-independent signaling in the development of an aging phenotype; however, which intracellular mechanisms are involved is not completely elucidated. Cdc42 [81,91], actin polarization [92], Ca^2+^ [81], and Notch proteins appear to be involved, but more studies are needed in this area to further clarify their relationships. Interestingly, the myeloid skewing and Cdc42 imbalance in HSCs submitted to cadmium exposure were also related to increased Wnt5a expression [99], underscoring the roles of Wnt5a in hematopoietic imbalances.

## 6. Hematopoietic Malignances

Hematological malignancies are disorders that include a diverse set of chronic and acute lymphoproliferative and myeloproliferative diseases, derived from the two major blood cell lineages: lymphoid and myeloid, respectively. For instance, myeloma, acute lymphocytic leukemia (ALL), chronic lymphocytic leukemia (CLL), and lymphoma are of lymphoid origin, whereas chronic myeloid leukemia (CML), acute myeloid leukemia (AML), and myelodysplastic syndrome (MDS) are of myeloid origin. The development of hematological malignancies requires malignant clones to lose their regulatory mechanisms, resulting in the production of a large number of dysfunctional cells and the disruption of normal hematopoiesis.

Most of the investigation about Wnt signaling and leukemogenesis focuses on β-catenin dependent signaling. There is evidence that β-catenin overexpression and its increased or constitutive activation drives leukemia initiation in some types of myeloid malignancies [89,100,101], which is associated with poor prognosis [102,103,104] and can be associated to Wnt3a or Wnt5a activity [105]. In fact, β-catenin is expressed in leukemic cell lines [86,106], whereas Wnt3a expression does not always follow the same profile [86].

In CML, whilst in vivo leukemic stem cells’ (LSC) survival was not affected by the inhibition of β-catenin signaling, it was in vitro [105], suggesting the involvement of other factors in a complex mechanism [107]. As a matter of fact, for CML, there is evidence that Wnt5a have β-catenin-dependent effects [105], whereas, in vivo, Wnt5a and DKK1 were described to act as proinflammatory agents and contribute to tumor suppression [78,108,109], hampering the understanding of Wnt5a in this context. 

Although most Wnt signaling research is focused on the Wnt/β-catenin pathway, interest in the independent branch is growing. In lymphoid leukemia, there is evidence that Wnt5a induces ROR1/ROR2 hetero-oligomerization and Rac1 activation [110], and that Wnt5a presence might offer a survival advantage [111]. This would not only activate β-catenin-independent signaling but would trigger the malignant process as well. Indeed, the worst prognosis is associated with CLL cells expressing high levels of Wnt5a [112]. Recently, Wnt5a was shown to induce ROR1 to activate cell proliferation and migration by binding to specific proteins [113]. Furthermore, in CLL, the ROR1 Pro-rich domain functions as a binding site for hematopoietic cell-specific Lyn substrate 1 (HS1), which undergoes tyrosine phosphorylation, also inducing migration [114]. The stimulation of the survival and proliferation of CLL cells through Wnt5a/ROR1/Rac1 [113] and Wnt5a/ROR1/PI3K/Akt [115,116] signaling pathways has been proposed. At this point, it is important to emphasize that the expression and role of Wnt components vary in the various CLL subgroups [117], but solutions with Wnt5a inhibition start to appear [118].

There is evidence pointing at Wnt5a as a therapeutic molecule against leukemias; by inhibiting β-catenin-dependent signaling, it would promote differentiation and reduce aberrant proliferation [59,101,119]. Remarkably, for ALL, no overexpression and even a downregulation of Wnt5a was observed [119,120]. 

Other studies in B-ALL and CLL models revealed a positive correlation between ROR1 expression and STAT3 activation [121,122]. Recently, Wnt5a/ROR1 signaling was also shown to increase the proliferation of B-cell precursor ALL (BCP-ALL) cells, with TCF3-PBX1 fusion gene expression via the activation of RhoA/Rac1 GTPases and STAT3 upregulation [123]. In another study, NFκB p65 was identified as a downstream target of Wnt5a/ROR1, leading to drug resistance in mantle cell lymphoma (MCL) [124]. Interestingly, drug sensitivity studies in t(1;19) B-ALL have demonstrated a great therapeutic efficacy of the Bcl-2 inhibitors venetoclax and navitoclax when combined with ROR1 targeting. This effect was confirmed in primary cells from a relapsed patient with TCF3-PBX-ALL [123]. Other studies also underscore that anti-ROR1 mAb treatment enhanced venetoclax activity in CLL and MCL [125], suggesting that, in ROR1-positive hematological cancers, this approach represents a promising strategy. Recently, the Wnt5a/ROR1 signaling was shown to induce NFκB-target gene expression in CLL cells, thus increasing IL-6 level, which, in turn, induces STAT3 activation [118]. In addition, the activation of STAT3 was inhibited by treatment with the anti-ROR1 mAb, cirmtuzumab. Importantly, anti-Wnt5a antibodies also circumvent the Wnt5a-dependent survival stimulus for CLL cells, thus blocking the survival advantage conferred by the microenvironment. Furthermore, a phase I clinical trial in CLL patients revealed that cirmtuzumab was also able to downregulate the expression of NFκB and STAT3-target genes in vivo [118]. It is worth mentioning that cirmtuzumab was also shown to inhibit Wnt5a-induced Rac1 activation in CLL in combination with ibrutinib, revealing a promising role for the anti-ROR1 mAb in blocking alternative survival signaling cascades, such as the BCR-signaling [126]. Together, these studies provide rationales for the inhibition of Wnt5a/ROR1 signaling in patients with leukemia.

Interestingly, a study of the gene expression pattern in AML revealed that cells exhibiting high levels of the epithelial cell adhesion molecule (EpCAM^+^) presented increased chemoresistance, which correlated with Wnt5b signaling activation. The study also demonstrated that the generation of anti-EpCAM antibodies promotes the effective elimination of AML cells through the immune system [127].

In addition, high levels of RYK expression were found in acute leukemia cell lines and patient samples, and mutations in genes encoding several Wnt components, including RYK, were suggested to affect the pathogenesis of CLL [117,128]. Interestingly, RYK was associated with PCP and Rho signaling in CLL patients. It was demonstrated that the Wnt/PCP proteins control chemotactic responses and the migratory ability of primary CLL cells after Wnt5a activation, and subsequent Rho activity [112,129,130].

## 7. Targeting Wnt Signaling: Therapeutic Opportunities

Studies defining the role of Wnt pathway components in the pathogenesis of hematological malignancies provide important insights for the development of new therapeutic strategies. In this respect, small molecule inhibitors of the Wnt/β-catenin pathway have been studied as potential new drugs to recognize intracellular targets [100,131]. Using the TOPFlash reporter assay, it was possible to screen small molecules able to inhibit the Wnt/β-catenin signaling with interesting results. Inhibitors of tankyrase 1 and 2, which lead to the stabilization of Axin and degradation of β-catenin, were identified in ALL [82]. In addition, another compound was identified to associate with CBP and compete for binding to β-catenin (ICG-001) in ALL and CML cells under hypoxic conditions [132,133,134].

Concerning the β-catenin independent branch of Wnt signaling, the expression of ROR1 almost exclusively in CLL cells and other lymphomas makes them relatively selective and attractive targets for the development of new antitumor drugs. In particular, monoclonal anti-ROR1 antibodies have been developed, of which UC-961, also known as cirmtuzumab, has entered phase I/II clinical trials (ID: NCT02222688) for CLL, after promising results were obtained in preclinical studies [135]. Recent studies demonstrated that this antibody was able to reduce CLL migration and proliferation, as well as tumor development, in mice, further supporting its clinical potential [110].

Chimeric antigen receptor T (CAR-T) cell therapy is another type of ROR1-targeted therapeutic approach that has been explored, with the advantage of low off-target cytotoxicity due to the aforementioned unique expression of ROR1 in cancer cells, such as CLL and MCL cells, but not in normal B cells [136]. This strategy also proved to be efficient in a xenograft mouse model of B cell lymphoma [137] and was well tolerated in primates in toxicological studies [138].

The effects of ROR1 inhibition have also been studied in other preclinical models, such as MCL, which expresses high levels of ROR1 as well. Studies using drug combinations revealed that inhibitors of several kinases, such as BTK (BCR pathway; ibrutinib, acalabrutinib), FAK (PF431396, VS-4718), and PKC (bryostatin 1, ponatinib) were more effective in killing sensitive MCL cells after shRNA-mediated ROR1 inhibition [124].

In contrast to ROR1, therapies able to target RYK have not yet been successfully demonstrated/developed. Recently, however, a monoclonal antibody with high affinity for the RYK WIF domain was developed by phage library, which could block Wnt5a signaling, and as such could hold promise for future therapies [139].

Despite the significant progress made over in recent decades in understanding the mechanisms by which Wnt signaling affects hematological malignancies, its full role in the pathogenesis of these diseases has not yet been completely established, mainly when considering the β-catenin independent branch and Wnt5 ligands. A deeper insight into the molecular mechanisms of Wnt signaling in hematological malignancies will pave the way for the development of innovative (combination) therapies in the near future.

## 8. Conclusions

Molecules involved in the modulation of hematopoiesis have been known for decades, but the introduction of Wnt signaling in this context changed how we understand this system, from early development to aging and disorders [109]. Wnt signaling and its crosstalk with well-known cytokines and other mediators further elucidate events such as HSC quiescence, proliferation, differentiation, and cell death. Wnt3a and associated β-catenin-dependent signaling have essential roles, such as expansion of hematopoietic-committed cells during ontogenesis, cell cycle entering of ST-HSC in adult subjects, and following lineage specification by differentiation, in addition to the modulation of lymphoid development. On the other hand, the β-catenin-independent pathway (widely associated with Wnt5a and b) is needed for hematopoietic initiation during embryonic development, LT-HSC quiescence maintenance and myeloid modulation in adult subjects, in addition to its involvement in aging-related hematopoietic alterations.

The ability to frequently rely on switches between β-catenin-dependent and -independent pathways suggests a highly interconnected system. In addition, multiple mechanisms of action of ligands, such as Wnt5a, further reinforce the idea of an interdependent system.

By now, the understanding of Wnt ligands in the hematopoietic system is under construction and, mainly when considering the β-catenin-independent pathway and the roles of Wnt5a and b in lineage specification and aging. Several points of attention still need to be addressed and elucidated, such as Wnt signaling crosstalk with other pathways involved in hematopoietic proliferation and differentiation (Ca^2+^, PI3K/AKT, STATs), extracellular molecules involved in Wnt signaling modulation (proteoglycans and extracellular matrix molecules) and interaction between growth factors and Wnt ligands, transcriptional targets of β-catenin-independent signaling, not to mention the mechanisms involved in Wnt-driven hematopoietic senescence, which is a high interest field. Aside from this, it may be of interest to focus on intermediate molecules (not only ligands and receptors), as it is undoubtable that the individual components involved in Wnt signaling are critically important in hematopoiesis, and that further understanding of the complex mechanisms involved in Wnt signaling and its relation to hematopoiesis, both in health and disease, will provide novel insights leading to future therapeutic strategies.

## Supplementary Materials

The following are available online at https://www.mdpi.com/2073-4409/9/8/1801/s1, Table S1: Surface markers used to characterize cells associated to initial steps of hematopoiesis.
