# Supplementary material for: Wnt-5A/B Signaling in Hematopoiesis throughout Life
Source: Cells. 2020 Jul 29;9(8):1801. doi: 10.3390/cells9081801 (PMC7465103; doi:10.3390/cells9081801)
Supplement: Supplementary file 1 [file cells-09-01801-s001.pdf]

Table S1. Surface markers used to characterize cells associated to initial steps of hematopoiesis.

Cell Type	Surface Markers	Reference
**LT-HSC**	Lin^{-} c-Kit^{+} Sca1^{+} CD34^{-} Flk2^{-} (mouse)	[19]
	CD201^{+} CD150^{+} CD48^{-} Lin^{-} c-Kit^{+} Sca-1^{+} (mouse)	Reviewed by Cheng 2020 [140]
	CD34^{+} Flk2^{-} Lin^{-} c-Kit^{+} Sca-1^{+} (mouse)	Reviewed by Cheng 2020 [140]
	Lin^{-} c-Kit^{+} Sca-1^{+} Flk2^{-} CD34^{-} Slamf1^{+} (mouse)	Reviewed by Chotinantakul 2012 [141]
	Lin^{-} CD34^{+} CD38^{-} CD90$^{+/-}$ CD45RA^{-} CD49f^{+} Rho^{low} (human)	Reviewed by Chotinantakul 2012 [141]
**ST-HSC**	CD34^{+} Flk2^{-} Lin^{-} c-Kit^{+} Sca-1^{+} (mouse)	Reviewed by Cheng 2020 [140]
	Lin^{-} c-Kit^{+} Sca-1^{+} Flk2^{-} CD34^{+} Slamf1^{+} (mouse)	Reviewed by Chotinantakul 2012 [141]
	Lin^{-} CD34^{+} CD38^{-} CD90$^{+/-}$ CD45RA^{-} CD49f^{+} Rho^{-} (human)	Reviewed by Chotinantakul 2012 [141]
**HSC (in general)**	CD34^{+} CD38^{-} Thy1^{-} CD45RA^{-} CD49f^{+} (human)	[17]
	CD34^{+}CD38^{-}Thy-1^{+}CD45RA^{-}Flt3^{+}CD7^{-}CD10^{-} (human)	[20]
**MPP**	CD34^{+} CD38^{-} Thy1^{-} CD45RA^{-} CD49f^{-} (human)	[17]
	CD34^{+} CD38^{-} Thy-1^{-} CD45RA^{-} Flt3^{+} CD7^{-} CD10^{-} (human)	[20]
	CD34^{+} Flk2^{+} Lin^{-} c-Kit^{+} Sca-1^{+} (mouse)	Reviewed by Cheng 2020 [140]
	CD150^{-} CD48^{-} Lin^{-} c-Kit^{+} Sca-1^{+} (mouse)	Reviewed by Cheng 2020 [140]
	Lin^{-} c-Kit^{+} Sca-1^{+} Flk2^{+} CD34^{+} Slamf1^{-} (mouse)	Reviewed by Chotinantakul 2012 [141]
**CLP**	CD34^{+} CD10^{+} CD24^{-} (human)	[142]
	Lin^{-} CD34^{+} CD38^{+} CD10^{+} (human)	Reviewed by Chotinantakul 2012 [141]
	Lin^{-} Flk2^{+} IL7Rα^{+} CD27^{+} (mouse)	Reviewed by Chotinantakul 2012 [141]
	Lin^{-} c-Kit^{low} Sca-1^{low} IL7Rα^{+} Flk2^{+} (mouse)	Reviewed by Cheng 2020 [140]
**CMP**	CD34^{+} CD38^{+} CD10^{-} FLT3^{+} CD45RA^{-} (human)	[17]
	CD34^{+} CD38^{+} Thy-1^{-} CD45RA^{-} Flt3^{+} CD7^{-} CD10^{-} (mouse)	[20]
	CD34^{+} CD38^{+} IL-3Rα^{+} CD45RA^{-} (human)	[143]
	CD34^{+} FcγRII/III^{low} Lin^{-} c-Kit^{+} Sca-1^{-} (mouse)	Reviewed by Cheng 2020 [140]
	Lin^{-} CD34^{+} CD38^{+} IL3Rα^{low} CD45RA^{-} (human)	Reviewed by Chotinantakul 2012 [141]
	Lin^{-} c-Kit^{+} Sca-1$^{-/low}$ CD34^{+} FcγR^{low} (mouse)	Reviewed by Chotinantakul 2012 [141]
